# Global profiling of protein lysine malonylation in *Toxoplasma gondii* strains of different virulence and genetic backgrounds

**DOI:** 10.1371/journal.pntd.0010431

**Published:** 2022-05-16

**Authors:** Lan-Bi Nie, Qin-Li Liang, Meng Wang, Rui Du, Meng-Yuan Zhang, Hany M. Elsheikha, Xing-Quan Zhu

**Affiliations:** 1 State Key Laboratory of Veterinary Etiological Biology, Key Laboratory of Veterinary Parasitology of Gansu Province, Lanzhou Veterinary Research Institute, Chinese Academy of Agricultural Sciences, Lanzhou, People’s Republic of China; 2 College of Animal Science and Technology, Jilin Agricultural University, Changchun, People’s Republic of China; 3 College of Veterinary Medicine, Shanxi Agricultural University, Taigu, People’s Republic of China; 4 Jingjie PTM Biolabs (Hangzhou) Co. Ltd., Hangzhou, People’s Republic of China; 5 Faculty of Medicine and Health Sciences, School of Veterinary Medicine and Science, University of Nottingham, Loughborough, United Kingdom; 6 Key Laboratory of Veterinary Public Health of Yunnan Province, College of Veterinary Medicine, Yunnan Agricultural University, Kunming, People’s Republic of China; Instituto de Salud Carlos III, SPAIN

## Abstract

Lysine malonylation is a post-translational modification (PTM), which regulates many cellular processes. Limited information is available about the level of lysine malonylation variations between *Toxoplasma gondii* strains of distinct genetic lineages. Yet, insights into such variations are needed to understand the extent to which lysine malonylation contributes to the differences in the virulence and repertoire of virulence factors between *T*. *gondii* genotypes. In this study, we profiled lysine malonylation in *T*. *gondii* using quantitative liquid chromatography-tandem mass spectrometry (LC-MS/MS) and immuno-affinity purification. This analysis was performed on three *T*. *gondii* strains with distinctive pathogenicity in mice, including RH strain (type I), PRU strain (type II), and VEG strain (type III). In total, 111 differentially malonylated proteins and 152 sites were upregulated, and 17 proteins and 17 sites were downregulated in RH strain versus PRU strain; 50 proteins and 59 sites were upregulated, 50 proteins and 53 sites were downregulated in RH strain versus VEG strain; and 72 proteins and 90 sites were upregulated, and 7 proteins and 8 sites were downregulated in VEG strain versus PRU strain. Differentially malonylated proteins were involved in key processes, such as those mediating the regulation of protein metabolism, stress response, glycolysis, and actin cytoskeleton. These results reveal an association between lysine malonylation and intra-species virulence differences in *T*. *gondii* and offer a new resource for elucidating the contribution of lysine malonylation to energy metabolism and virulence in *T*. *gondii*.

## Author summary

Lysine malonylation has been shown to play important roles in various biological processes in *Toxoplasma gondii*. Here, we used quantitative liquid chromatography-tandem mass spectrometry (LC-MS/MS) and immuno-affinity purification to test the hypothesis that lysine malonylation underpins the inter-genotype differences in the virulence of *T*. *gondii*. Several up-regulated and down- regulated malonylated proteins were identified in the tachyzoites of RH (type I) strain, PRU (type II) strain, and VEG (type III) strain. Differentially regulated malonylated proteins were enriched in many biological and metabolic pathways, and were found to contribute *T*. *gondii* energy metabolism, stress response, and infectivity, suggesting the role of lysine malonylation in the regulation of *T*. *gondii* virulence. These findings expand our knowledge of lysine malonylation in *T*. *gondii* and provide more insight into the mechanisms mediating the virulence differences between *T*. *gondii* strains of different genotypes.

## Introduction

The obligate intracellular parasite *Toxoplasma gondii* infects a wide range of warm-blooded animals and is highly prevalent in humans [1,2]. This parasite imposes a significant risk to patients with a compromised immune system and to pregnant woman [3–5]. *T*. *gondii* strains are grouped into three genetically distinct lineages, known as types I, II, and III, which vary significantly in virulence in mice [6,7]. Besides these three established genotypes, atypical *T*. *gondii* genotypes have been reported, such as Chinese I (ToxoDB 9) in China [8–10], Africa 1 and Africa 3 in Africa, and type 12 in North American wild animals [11].

The ability of *T*. *gondii* to establish an infection relates to the expression of a wide range of virulence factors. These effector proteins play important roles in promoting the parasite invasion and colonization of host cells, and evasion of innate and adaptive immune responses. Early transcriptomic and proteomics studies have shown that virulence factors produced by *T*. *gondii* vary between different clonal lineages [12,13], possibly attributed to the plasticity in proteins required for parasite invasion [14].

Lysine malonylation is a type of protein post-translational modification (PTM) reported in eukaryotes and bacteria [15,16]. The regulatory role of lysine malonylation in many biological processes has been established in various organisms [17–19]. Lysine malonylation affects energy metabolism, mitochondrial function, and fatty acid synthesis [20]. However, this type of PTM remains poorly understood in *T*. *gondii*, with only one study having investigated its expression pattern in one *T*. *gondii* strain [21].

It is unclear what regulatory mechanism lysine malonylation mediates in *T*. *gondii* and whether malonylation contributes to the genotype-related differences in the virulence of *T*. *gondii*. Recent studies have suggested a role for lysine acetylation in the virulence *T*. *gondii* strains of different genotypes [22,23]. Therefore, it is reasonable to hypothesize that lysine malonylation contributes to the proteomic differences between *T*. *gondii* strains of different genotypes, especially in proteins related to virulence and pathogenicity.

Here, we used liquid chromatography-tandem mass spectrometry with immuno-affinity purification to investigate the differences in lysine malonylation between *T*. *gondii* strains of different genotypes, including RH strain (type I), PRU strain (type II), and VEG strain (type III). Our data provide new insight into the role of lysine malonylation in the genotypic differences in *T*. *gondii* virulence.

## Materials and methods

### Cell and parasite culture

Tachyzoites of three *T*. *gondii* strains, RH strain (type I), PRU strain (type II) and VEG strain (type III), were used in this study. All strains were maintained by serial passage in human foreskin fibroblast (HFF) cells originally obtained from American Type Culture Collection (ATCC, Manassas, VA, USA). HFFs were grown in Dulbecco’s modified Eagle medium (DMEM) supplemented with 10% fetal bovine serum (FBS, Gibco, USA), 100 U/ml antibiotics (penicillin-streptomycin solution). The infected cell cultures were incubated at 37°C with 5% CO_2_. Tachyzoites were separated from the feeder host cells by passage through 25-gauge syringe needles. A 3 μm membrane filter (Millipore) was used to remove the cell debris, and the tachyzoites were washed with phosphate-buffered saline (PBS) and centrifuged at 2,000 × *g* twice. Purified tachyzoite pellets were stored at –80°C until use.

### Protein extraction

To extract total protein from RH, PRU and VEG strains, ~ 3 × 10^9^ tachyzoites of each strain were transferred from –80°C freezer and thawed at room temperature. Lysis buffer (1% dodecyl sulfate, sodium salt [SDS], 1% protease inhibitor cocktail, 5 mM dithiothreitol [DTT], 3 μM trichostatin A [TSA] and 50 mM nicotinamide [NAM]) was added to tachyzoites and the crude lysate was sonicated three times on ice (220 W, sonicated 3 seconds, paused for 5 seconds, and repeat three times). The samples were centrifuged at 2,000 ×*g* for 10 min at 4°C to remove the cell debris. The clear supernatant was collected and transferred to a new centrifuge tube and stored at –80°C. Bradford protein assay (Bio-Rad Laboratories, Hercules, CA) was used to determine the protein concentration in the supernatant.

### Trypsin digestion

An equal quantity of 200 μg of protein from each sample was prepared. Each biological replicate was analyzed in three technical triplicates. Trichloroacetic acid was slowly added to each sample to a final concentration of 20%, and the sample was mixed by vortexing and precipitated at 4°C for 2 h. The protein pellet was obtained by centrifugation at 4, 500 ×*g* for 5 min, the supernatant was discarded, and the precipitate was washed with chilled acetone for 2–3 times. After the protein pellets were air-dried, 200 mM triethylammonium bicarbonate (TEAB) was added to each sample to resuspend the protein pellet by ultrasound sonication, and then the trypsin was added at a ratio of 1:50 (protease: protein, M/M) overnight. DTT was added to a final concentration of 5 mM. Protein reduction was performed at 56°C for 30 min followed by alkylation by adding iodoacetamide (IAA) at a final concentration of 11 mM and incubation for 15 min in the dark at room temperature.

### Modification enrichment

After trypsin digestion, resulting peptides were dissolved in IP buffer solution (100 mM NaCl, 1 mm EDTA, 50 mM Tris HCl, 0.5% NP-40, pH 8.0). The supernatant was transferred to a pre-washed pan anti-malonyllysine antibody resin (No. PTM-904, PTM Bio, Hangzhou). The peptide solution and antibody bead mixture were placed overnight on a shaker at 4°C. After incubation, the resin was washed with IP buffer solution four times and twice with deionized water. Finally, the resin bound peptides were eluted with 0.1% trifluoroacetic acid for three times. The eluent was collected and vacuum dried. After drying, peptides were desalted using C18 ziptips, and the clean peptides were vacuum dried for LC-MS/MS analysis.

### LC-MS/MS analysis

Enriched peptides were dissolved in liquid chromatography mobile phase A and separated by ultra-performance liquid chromatography (UPLC). The mobile phase A was aqueous solution containing 0.1% formic acid and 2% acetonitrile, the mobile phase B was aqueous solution containing 0.1% formic acid and 100% acetonitrile. The gradient involved an increase from 6 to 23% solvent B (0.1% formic acid in 98% acetonitrile) over 26 min, 23 to 35% in 8 min, and climbing to 80% in 3 min and then holding at 80% for the last 3 min. The peptides were separated by UPLC system, then ionized by capillary ion source and analyzed by tims-TOF Pro mass spectrometer. The ion source voltage was set at 2.0 kV, peptide parent ion and its secondary fragments were detected and analyzed by high-resolution TOF mass analyzer. The scanning range of secondary mass spectrometry was set at 100–1700. The data acquisition mode was PASEF. After a first-order mass spectrometer collected, 10 times of the PASEF mode was used to collect the second-order spectrum with the charge number of parent ions in the range of 0–5. The dynamic exclusion time of tandem mass spectrometry scanning was set to 30 seconds to avoid repeated scanning of parent ions.

### Database search

Raw mass spectrometry data was searched against *T*. *gondii* database ToxoDB 48 (8,322 sequences) using MaxQuant (1.6.15.0) software. A reverse library was added to calculate the false discovery rate (FDR) caused by random matching, and common contamination library was added to the database to eliminate contaminated protein in the identification results. Enzyme digestion method was set to trypsin/P, number of missing cut sites of 4, and minimum length of peptide segment of 7 amino acid residues. Maximum modification number of peptide segment was 5. Mass error was set at 0 ppm and 20 ppm for the primary parent ion of search and main search, and 20.0 ppm for the secondary fragment ion. Peptide quantification was performed using label free quantification (LFQ) model in MaxQuant, FDR of protein identification and PSM identification was set at 1%.

### Bioinformatics analysis

Gene Ontology (GO) annotation of the proteome was based on the UniProt-GOA (http://www.ebi.ac.uk/GOA/) and ToxoDB 48 database. Briefly, protein ID was converted to UniProt ID and UniProt ID was matched to GO ID. Then, the corresponding information was extracted from UniProt-GOA database based on GO ID. In the case of absence of protein information in UniProt GOA database, InterProScan was used to predict the GO function of the protein using an algorithm based on protein sequence. The identified proteins were classified according to cell composition, molecular function, and physiological process. InterProScan based on protein sequence and the corresponding InterPro (http://www.ebi.ac.uk/interpro/) were used to annotate the protein domain. Online service tool KEGG Automatic Annotation Server (KAAS) of Kyoto Encyclopedia of Genes and Genomes (KEGG) was used to annotate the submitted proteins, and then KEGG mapper was used to match the annotated proteins to the corresponding pathways in the database. The wolfpsort (https://wolfpsort.hgc.jp/) was used to annotate the subcellular localization of the eukaryotic proteins.

Fisher’s exact test was used to detect differentially expressed malonylated proteins of GO and KEGG annotation. *P*-value < 0.05 was considered significant. A fold change > 1.5 was considered the threshold of significantly upregulated and downregulated proteins, respectively. InterPro (http://www.ebi.ac.uk/interpro/) was used to analyze the enrichment of the functional domains of differentially expressed proteins. The selected *P*-value matrix was transformed by − log10 and the hierarchical clustering (Euclidean distance, average connection clustering) method was used for one-sided clustering analysis. The clustering relationship was visualized by heat map constructed using the function Heatmap. 2 in R language package gplots. MoMo (http://meme-suite.org/tools/momo) was used to analyze the motif characteristics of the modification sites. When the number of peptides in a specific sequence is more than 20 and *P*-value < 0.000001, the specific sequence was considered a motif of a modified peptide. Differentially expressed malonylated proteins screened from different strain comparison groups were mapped into protein-protein interaction (PPI) network database of STRING (v.10.5) (http://string-db.org/) and the protein interaction relationship was extracted according to a confidence score > 0.7. R package "network D3" was used to visualize the PPI network.

## Results

### Identification of the differentially expressed proteins

**Fig 1:**
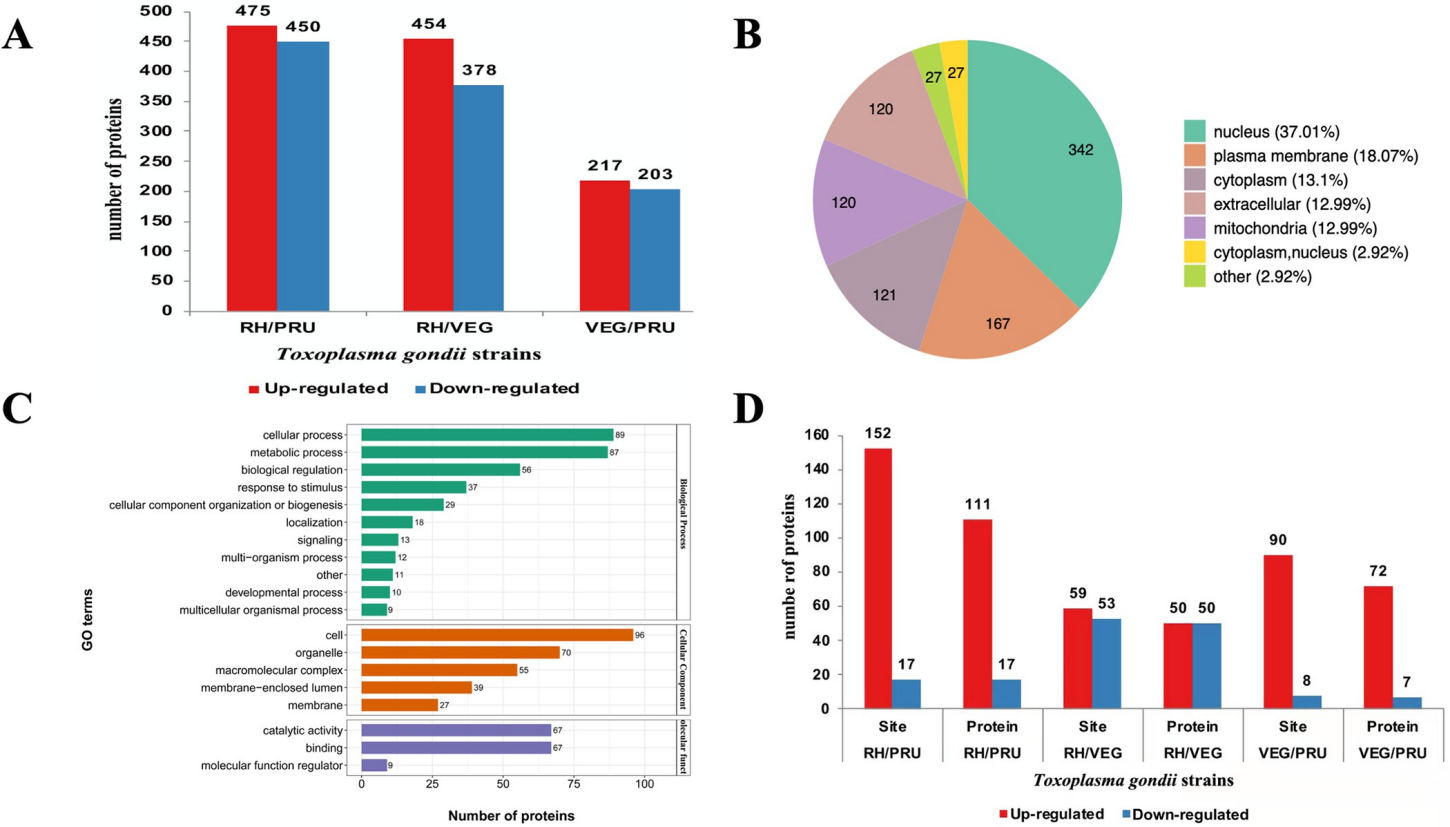
The identification of differentially expressed proteins in *Toxoplasma gondii*. **(A)** The number of differentially expressed proteins between the three *T*. *gondii* strains (RH, PRU, VEG). **(B)** The subcellular locations of the differentially expressed proteins. **(C)** Gene Ontology (GO) functional annotation of the differentially expressed proteins. **D)** The number of differentially expressed malonylated proteins between the three *T*. *gondii* strains.

Our analysis identified 3,920 proteins in all *T*. *gondii* strains, of which 3,534 proteins were quantifiable. We also identified 3,486, 3,473, and 3,504 proteins in RH, PRU and VEG, respectively (S1 Table). By comparing RH strain to PRU strain, 475 and 450 proteins were found upregulated and downregulated, respectively. Regarding RH strain vs. VEG strain, 454 and 378 proteins were upregulated and downregulated, respectively. For VEG strain vs. PRU strain, 217 and 203 proteins were upregulated and downregulated, respectively (Fig 1A).

The differentially expressed proteins in RH, PRU and VEG strains were detected in the nucleus (37.01%), plasma membrane (18.07%), cytoplasm (13.10%), extracellular (12.99%), and mitochondria (12.99%) (Fig 1B). GO annotation indicated that for biological process (BP) category, 89, 87, 56, and 37 proteins were identified in cellular process, metabolic process, biological regulation, and response to stimulus, respectively. For cellular component (CC) category, 96, 70, 55 and 39 proteins were enriched in cell, organelle, macromolecular complex, and membrane-enclosed lumen, respectively. Regarding the molecular function (MF) category, 67 proteins were involved in catalytic activity and binding (Fig 1C).

### Differentially malonylated proteins between *T*. *gondii* strains

To gain more insight into differences in lysine malonylation between *T*. *gondii* genotypes, LC-MS/MS and bioinformatic analysis were used to analyze the enriched malonylated peptides and sites. Lysine-malonylated peptides were enriched by immuno-affinity using the protein extracted from tachyzoites of RH (type I) strain, PRU (type II) strain and VEG (type III) strain. A total of 44,2496 secondary spectra were obtained. After searching the protein data library for the secondary spectra, the number of available effective spectra was 52,159 and the spectrum utilization rate was 11.8%. A total of 9,525 peptides were identified, of those 1,812 were identified as malonylated peptides from 775 proteins; of which 510 proteins and 1,119 sites were quantifiable (S2 Table). Of those, 111 proteins and 152 sites were up-regulated, and 17 proteins and sites were down-regulated in RH vs. PRU strains; 50 proteins and 59 sites were up-regulated, and 50 proteins and 53 sites were down-regulated in RH vs VEG strains; and 72 proteins and 90 sites were up-regulated, and 7 proteins and 8 sites were down-regulated in VEG vs. PRU strains (Fig 1D).

### Motif analysis of malonylated sites in RH, PRU and VEG strains

**Fig 2:**
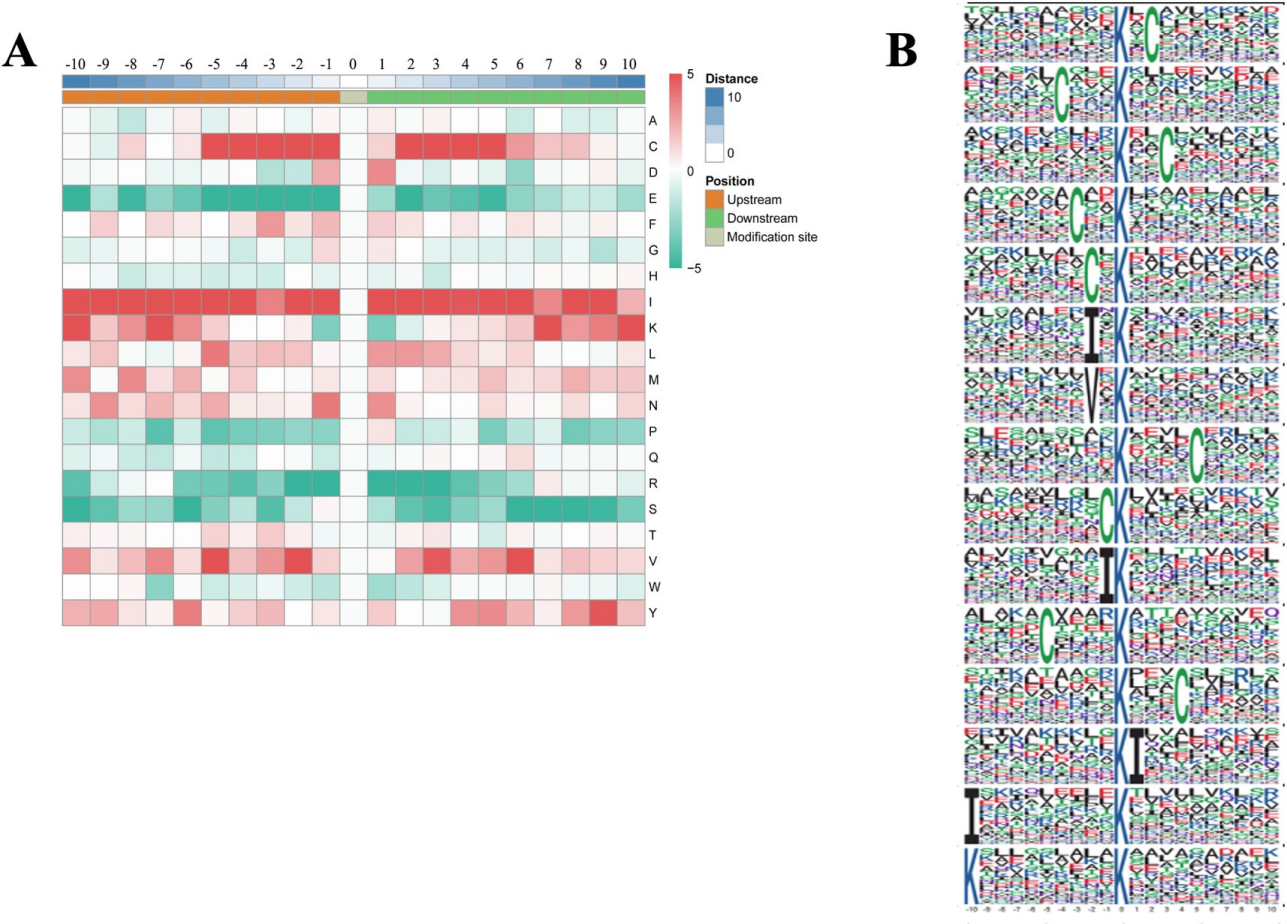
**(A)** Heat map of the different types of amino acids at positions −10 to +10 from the malonylated lysine residue. Red and green colors denote high and low frequency, respectively. **(B)** Sequence motif logos showing the malonylation sites detected in proteins and the position-specific amino acids’ composition surrounding the malonylation sites.

MoMo software and hierarchical cluster analysis were used to study the malonylated sites from 10 amino acids upstream to 10 amino acids downstream the flanking sequences. The frequency of cysteine (C) residue at the position -5 to -1 and +2 to +5 was highest, isoleucine (I) residue was enriched at -10 to +10, lysine (K) residue was enriched at -10 to -7 and +7 to +10, valine (V) residue was highest at -5, -2, +3 and +6 position, tyrosine (Y) residue was mainly enriched at +9 position. Glutamic acid (E), proline (P), arginine (R) and serine (S) were underrepresented in most positions (Fig 2A). Red color indicates that amino acid is significantly enriched near the modification site, while green color indicates that amino acid is significantly reduced near the modification site. In total, 15 motifs were identified, including K_mal_ X_1_ C, C X_3_ K_mal_, K_mal_ X_2_ C, C X_2_ K_mal_, C X K_mal_, I X K_mal_, V X K_mal_, K_mal_ X_4_ C, C K_mal_, I K_mal_, C X_4_ K_mal_, K_mal_ X_3_ C, K_mal_ I, I X_9_ K_mal_ and K X_9_ K_mal_ (Kmal indicates the lysine malonylated site, X represents a random amino acid residue) (Fig 2B).

### Functional enrichment of differentially malonylated proteins in RH and PRU strains

**Fig 3:**
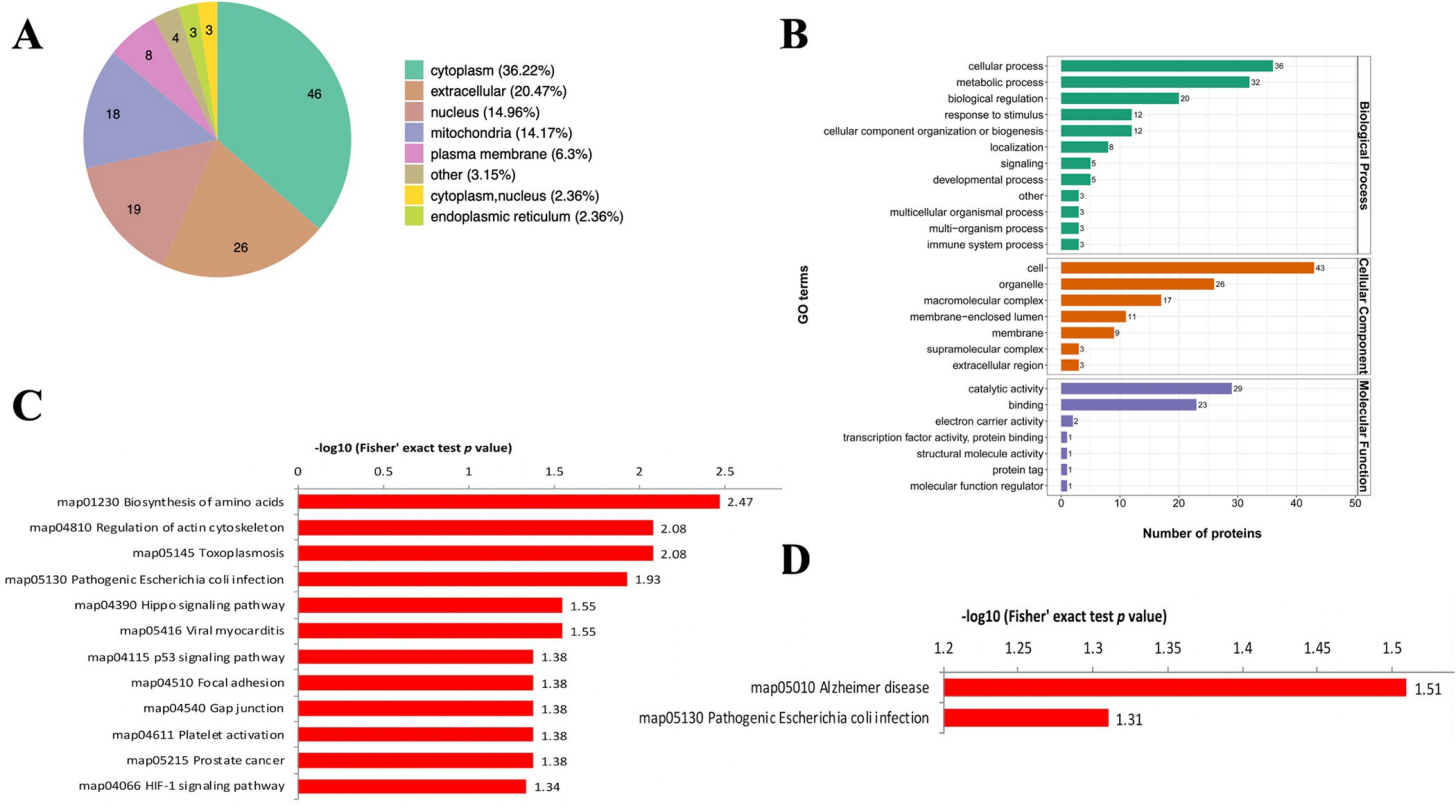
Functional enrichment analysis of the differentially malonylated proteins in RH vs. PRU strains. **(A)** Subcellular location of differentially lysine malonylated proteins in *T*. *gondii*. **(B)** Primary GO enrichment analysis of the differentially malonylated proteins. **(C)** KEGG pathway enrichment of differentially malonylated proteins. **(D)** KEGG pathway enrichment of the downregulated malonylated proteins.

Identification of subcellular location indicated that 36.22% differentially malonylated proteins in RH and PRU strains are localized in the cytoplasm, 20.47% are extracellular, 14.96% in the nucleus and 14.18% in the mitochondria (Fig 3A). Primary GO enrichment analysis of the differentially malonylated proteins at the second level showed that for the BP category, 36, 32, 20, and 12 differentially malonylated proteins were enriched in cellular process, metabolic process, biological regulation, and response to stimulus. For the CC category, 43, 26, 17, 11 and 9 malonylated proteins were enriched in cell, organelle, macromolecular complex, membrane-enclosed lumen, and membrane. Regarding the MF category, proteins were mainly enriched in catalytic activity (29 proteins) and binding (23 proteins) (Fig 3B).

**Fig 4:**
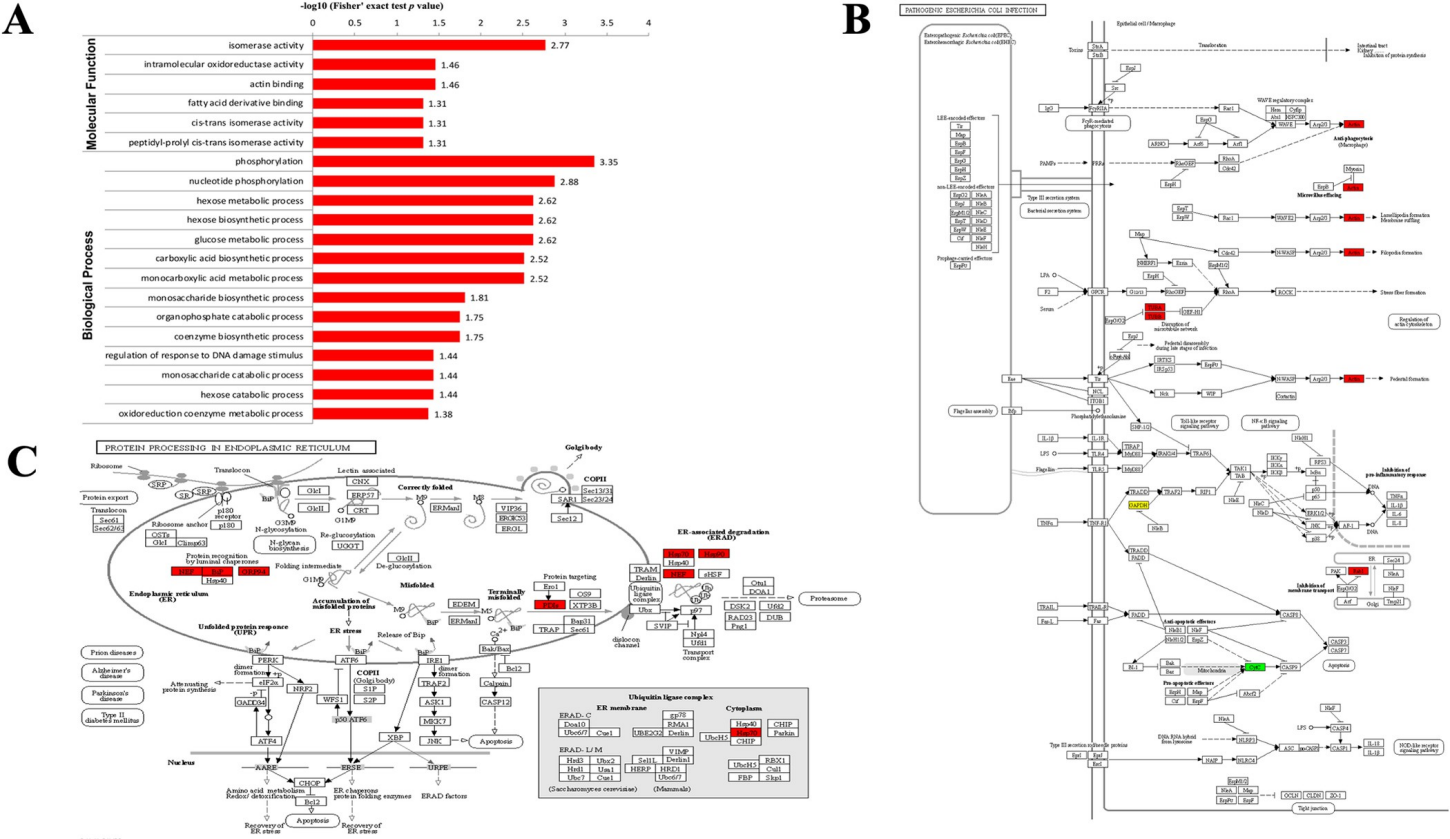
Functional enrichment analysis of differentially malonylated proteins in RH vs. PRU strains. **(A)** Advanced GO enrichment of the differentially malonylated proteins. **(B)** Alterations in the pathogenic *Escherichia coli* infection pathway with significantly enriched modified proteins showing inside red boxes. **(C)** Alterations in protein processing in endoplasmic reticulum pathway with significantly enriched modified proteins showing inside red boxes.

KEGG pathway enrichment analysis indicated that the differentially malonylated proteins were significantly enriched in biosynthesis of amino acids, regulation of actin cytoskeleton, toxoplasmosis, and pathogenic *Escherichia coli* infection (Fig 3C). The down-regulated proteins were enriched in Alzheimer disease and pathogenic *Escherichia coli* infection (Fig 3D). Advanced GO enrichment showed that in the MF category, malonylated proteins were enriched in GO terms related to isomerase activity, intramolecular oxidoreductase activity, and actin binding. In the BP category, proteins were mainly enriched in phosphorylation, nucleotide phosphorylation, hexose metabolic process, hexose biosynthetic process, and glucose metabolic process (Fig 4A). We found seven differentially malonylated proteins involved in protein processing in endoplasmic reticulum and four proteins involved in pathogenic *Escherichia coli* infection (Fig 4B and 4C).

### Functional enrichment of differentially malonylated proteins in RH and VEG strains

**Fig 5:**
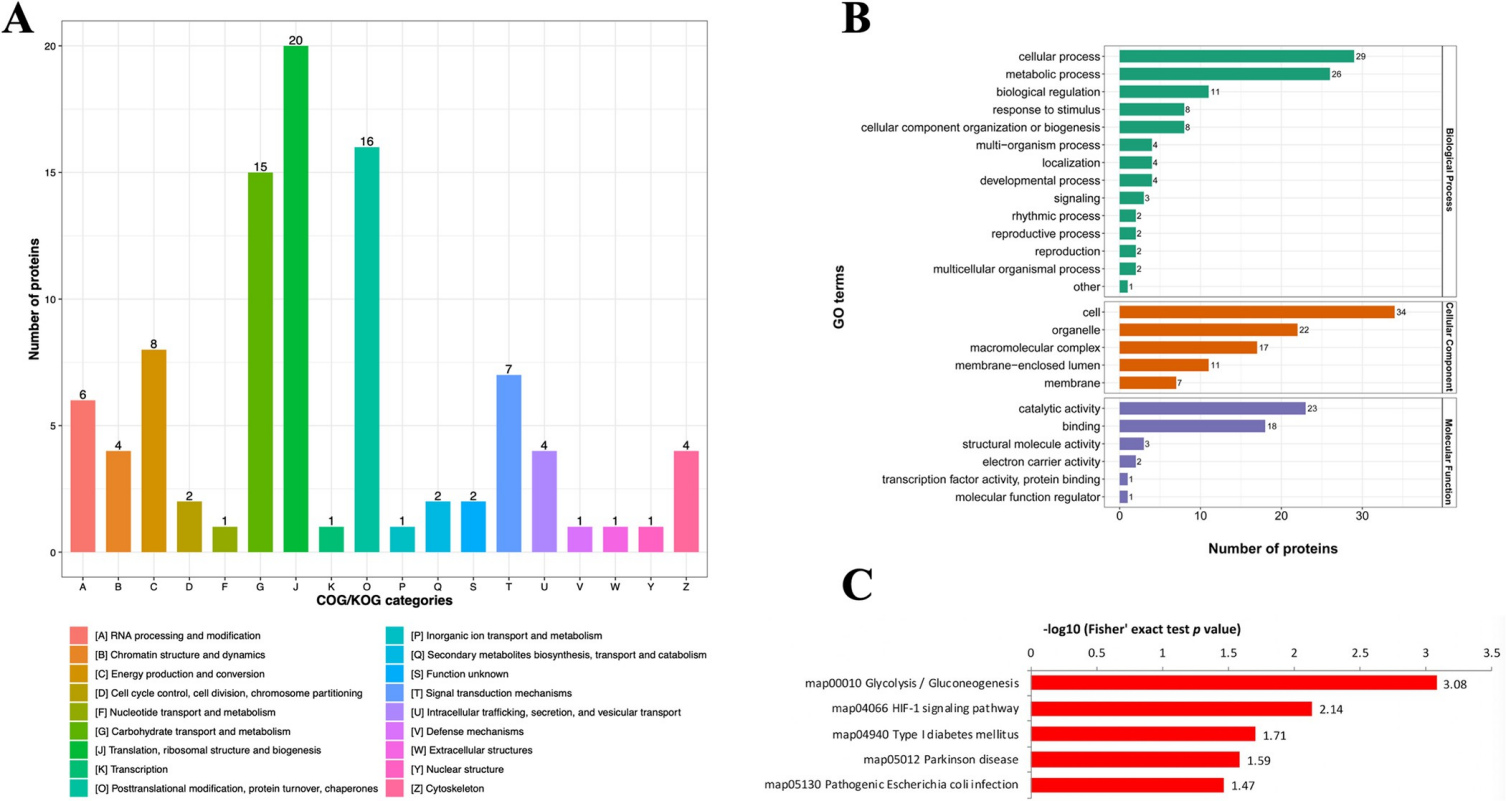
Functional enrichment analysis of differentially malonylated proteins in RH vs. VEG strains. **(A)** COG/KOG analysis of the differentially malonylated proteins. **(B)** GO classification and **(C)** KEGG pathway enrichment analysis of the differentially malonylated proteins.

**Fig 6:**
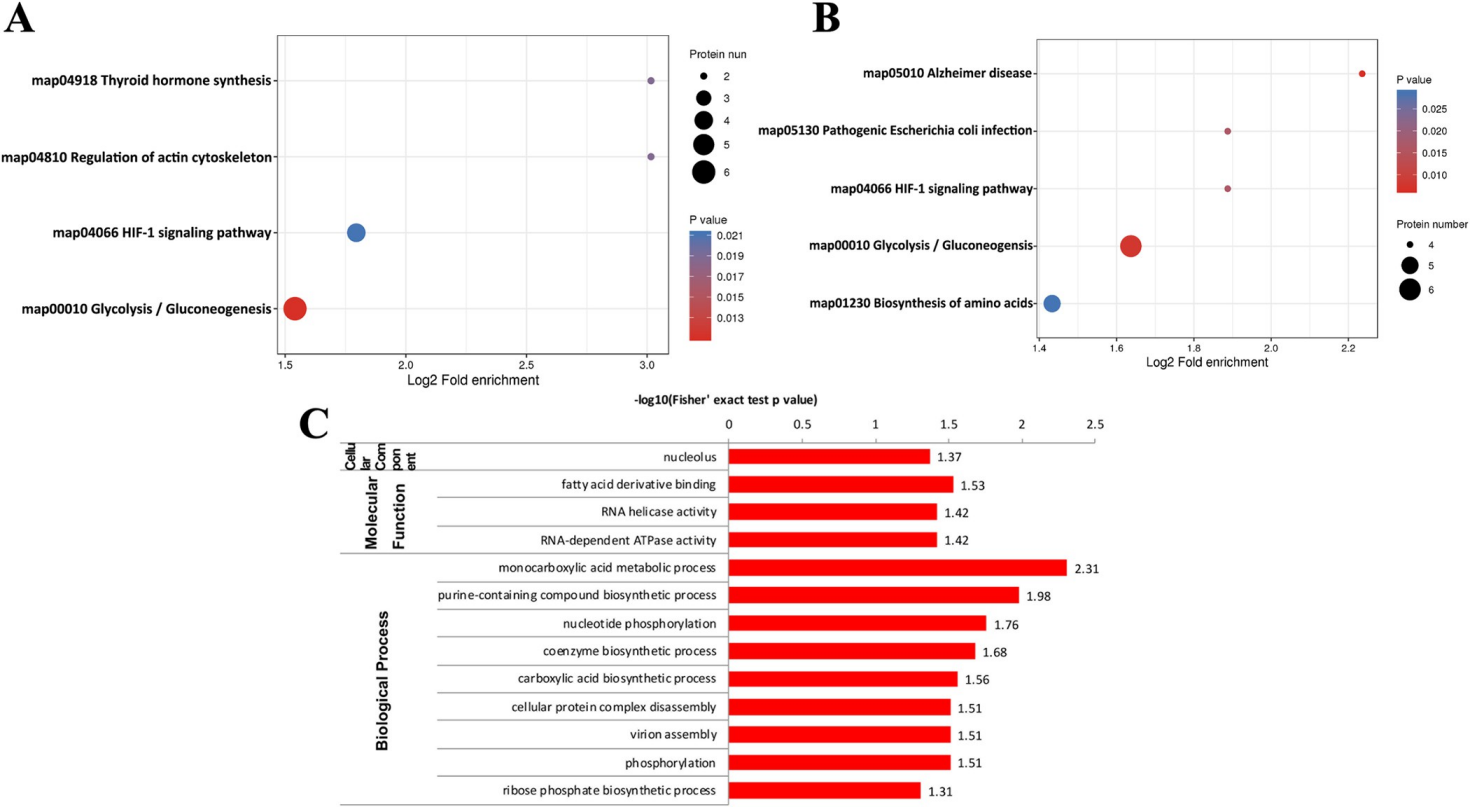
Functional enrichment analysis of the differentially malonylated proteins in RH vs. VEG strains. **(A)** KEGG pathway enrichment analysis of the **(A)** up-regulated and **(B)** down-regulated malonylated proteins. **(C)** GO enrichment of the differentially malonylated proteins.

We investigated the biological function of the differentially modified proteins in RH and VEG strains. As shown in Fig 5A, the differentially malonylated proteins were clustered in the GO terms of translation, ribosomal structure and biogenesis, posttranslational modification, protein turnover, chaperones and carbohydrate transport, and metabolism. GO classification analysis demonstrated that in BP category, most differential proteins in RH and VEG were enriched in cellular process, metabolic process, and biological regulation. For the CC category, 34 differential proteins between RH and VEG strains were detected in the cell, 22 proteins were in organelle and 17 proteins in macromolecular complex. For the MF category, 23 and 18 proteins were involved in catalytic activity and binding, respectively (Fig 5B). The biological functions of the differentially malonylated proteins detected in RH and VEG strains were similar to those of RH and PRU. The result of KEGG pathway enrichment revealed that differentially malonylated proteins were mainly enriched in glycolysis/ gluconeogenesis, HIF-1 signaling pathway, type I diabetes mellitus, Parkinson disease and pathogenic *Escherichia coli* infection (Fig 5C and S3 Table); Likewise, the up- and down- regulated proteins were mainly enriched in glycolysis/gluconeogenesis (Fig 6A and 6B). Furthermore, GO enrichment analysis showed that most of the differential proteins were enriched in nucleolus in the CC category, fatty acid derivative binding, RNA helicase activity and RNA-dependent ATPase activity in the MF category, and monocarboxylic acid metabolic process, purine-containing compound biosynthetic process and nucleotide phosphorylation in the BP category (Fig 6C).

### Functional enrichment of differentially malonylated proteins in PRU and VEG strains

**Fig 7:**
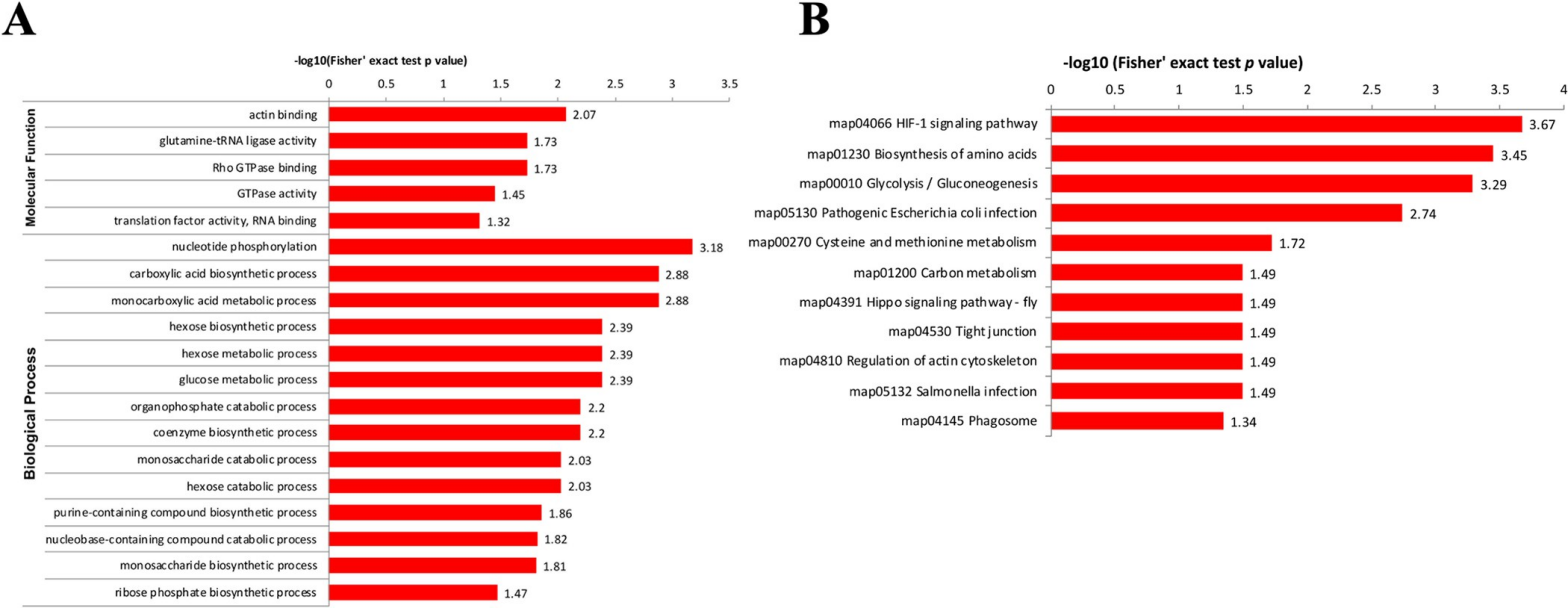
Functional enrichment analysis of the differential malonylated proteins in PRU vs. VEG strains. **(A)** GO analysis and **B)** KEGG pathway analysis of the differentially malonylated proteins.

GO analysis was used to annotate the function of the differentially malonylated proteins in PRU and VEG strains. The enriched GO terms were related to actin binding, glutamine-tRNA ligase activity and Rho GTPase binding in MF category; nucleotide phosphorylation, carboxylic acid biosynthetic process and monocarboxylic acid metabolic process were enriched in the BP category (Fig 7A and S4 Table). KEGG pathway showed that proteins were enriched in HIF-1 signaling pathway, biosynthesis of amino acids, glycolysis/gluconeogenesis, and pathogenic *Escherichia coli* infection (Fig 7B).

### Functional cluster of the differentially malonylated proteins in RH, PRU and VEG strains

**Fig 8:**
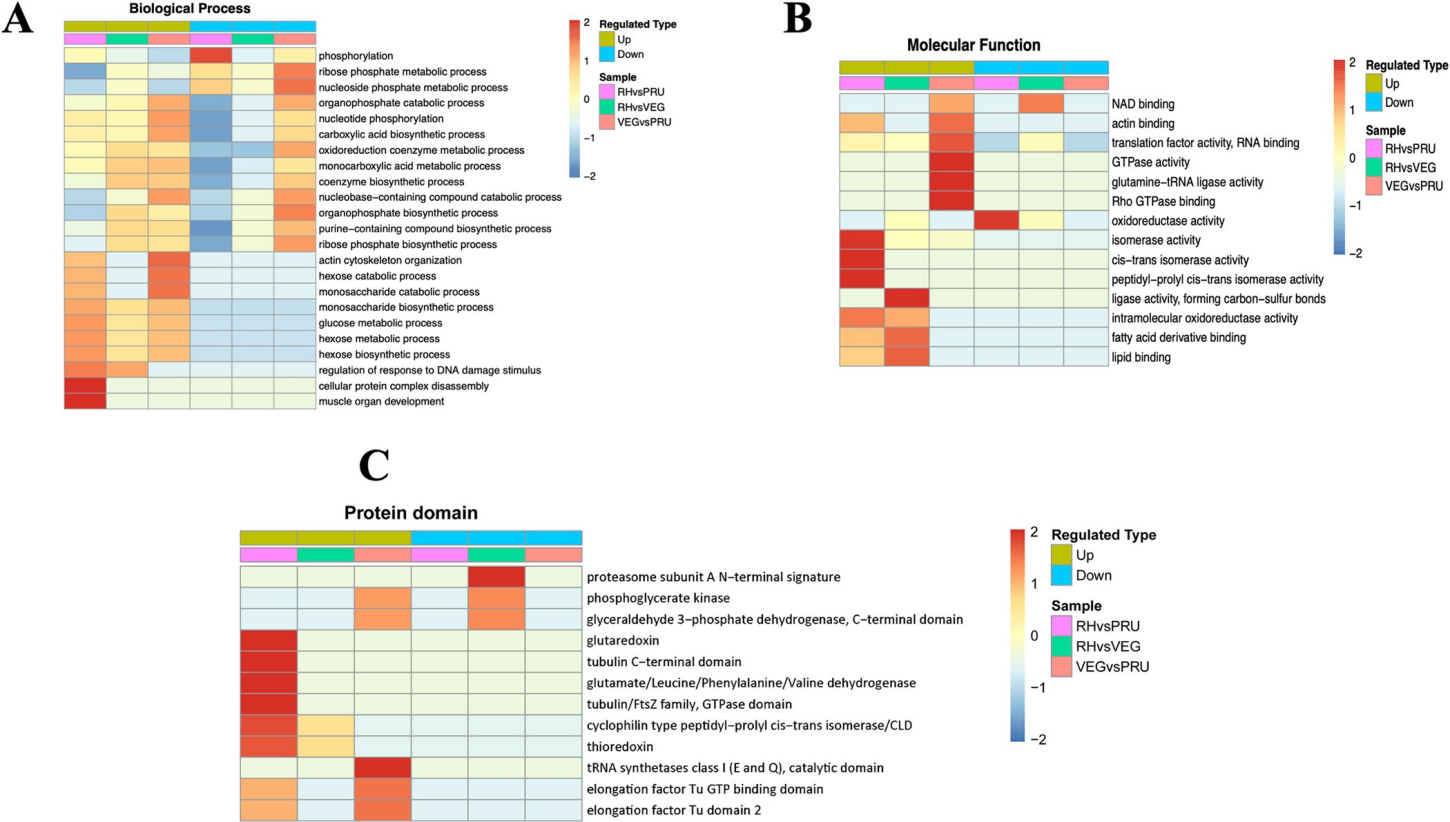
Functional clustering of the differentially malonylated proteins in RH, PRU and VEG strains. **(A)** Biological process. **(B)** Molecular function. **(C)** protein domain.

To obtain more insight into the function of up-regulated and down-regulated proteins, functional enrichment cluster was performed. Some important biological processes were enriched in the up-regulated and down-regulated proteins. By comparing RH vs. PRU strains, most up-regulated proteins were enriched in cellular protein complex disassembly and muscle organ development, and the down-regulated proteins were enriched in phosphorylation. In RH vs. VEG, the upregulated and downregulated proteins were enriched in the response to DNA damage stimulus and oxidoreduction coenzyme metabolic process. The upregulated proteins in VEG vs. PRU strains were enriched in actin cytoskeleton organization, hexose catabolic process and monosaccharide catabolic process, while the downregulated proteins were enriched in ribose phosphate metabolic process and nucleoside phosphate metabolic process (Fig 8A). In the molecular function category, the up-regulated proteins in RH vs. PRU strains were enriched in GO terms related to isomerase activity, cis-trans isomerase activity and peptidyl-prolyl cis-trans isomerase activity, while the down-regulated proteins were only enriched in oxidoreductase activity. The upregulated proteins in RH vs. VEG strains were mainly enriched in ligase activity, forming carbon-sulfur bonds, while the upregulated PRU vs. VEG malonylated proteins were enriched in GTPase activity, glutamine-tRNA ligase activity and Rho GTPase activity (Fig 8B). Additionally, protein domain results demonstrated that for RH vs. PRU strains, the up-regulated proteins were clustered in glutaredoxin, tubulin C-terminal domain, glutamate/leucine/phenylalanine/valine dehydrogenase and tubulin/FtsZ family, GTPase domain. The upregulated proteins in PRU vs. VEG strains were clustered in tRNA synthetases class I, catalytic domain. However, the down-regulated proteins in RH vs. VEG strains were clustered in proteasome subunit A N-terminal signature (Fig 8C).

### Protein-protein interaction (PPI) networks of the malonylated proteins

**Fig 9:**
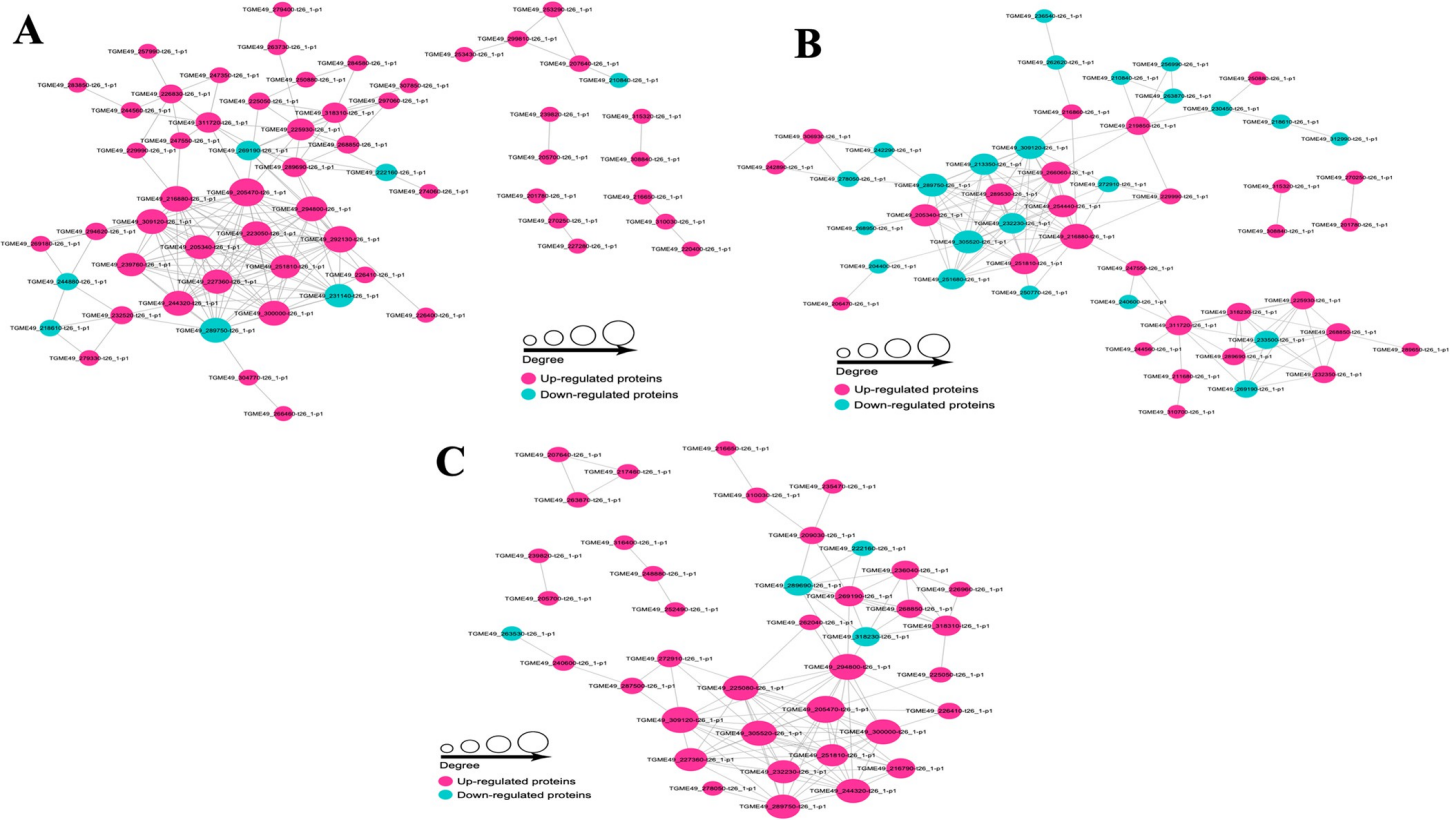
Protein-protein interaction (PPI) networks of the differential malonylation proteins. Cytoscape software and String database were used to construct the PPI networks of the differentially malonylated proteins in **(A)** RH vs. PRU, **(B)** RH vs. VEG, and **(C)** VEG vs. PRU. Nodes represent differentially expressed proteins. Size of the node represents number of the differential proteins and their interacting proteins. The larger the node, the more proteins it interacts with, indicating that the protein is more important in the network. Cyan and purple colors represent low and high expression of the corresponding proteins.

A total of 61 nodes were identified in PPI network, and 54 were up-regulated proteins and seven were down-regulated proteins in RH vs. PRU (Fig 9A). Nodes with a high degree of interaction with other proteins were defined as ‘hub’ proteins. Significant hub proteins identified include, for example translation elongation factor 2 family protein, guanine nucleotide-binding protein, ribosomal protein (RPL13A), elongation factor 1-alpha (EF-1-ALPHA), and ribosomal protein (RPL4). Fifty-one protein nodes were detected in RH vs. VEG, the most significant protein was guanine nucleotide-binding protein (Fig 9B). In VEG vs. PRU, 40 nodes were identified, of these 36 proteins were up-regulated, among which the most important proteins are translation elongation factor 2 family protein, elongation factor 1-alpha (EF-1-ALPHA), ribosomal protein (RPS18), ribosomal protein (RPL4), and ribosomal protein (RPL18) (Fig 9C).

## Discussion

We investigated a possible basis for lysine malonylation in the differences in the virulence between *T*. *gondii* strains of different genetic backgrounds. We examined three genetically distinct strains of *T*. *gondii* that differ in their virulence for mice: RH virulent strain (type I), PRU less virulent strain (type II), and VEG strain (type III), which is avirulent. By comparing the virulent RH strain with the less virulent PRU strain, we identified 17 down-regulated malonylated proteins, such as calcium-dependent protein kinase 1 (CDPK1) and ribosomal-ubiquitin protein (RPL40). The CDPK1 belongs to the serine/threonine kinase family which plays roles in the motility, organelle secretion, cell invasion, and egress of *T*. *gondii* [24]. Chemical inhibition of CDPK1 reduces *T*. *gondii* growth *in vitro*, reduces parasite dissemination to the central nervous system in mice, and inhibits reactivation of latent infection in immunocompromised mice [25]. RPL40 is an essential virulence factor and may play a role in the pathogenesis of acute *T*. *gondii* infection [26].

**Fig 10:**
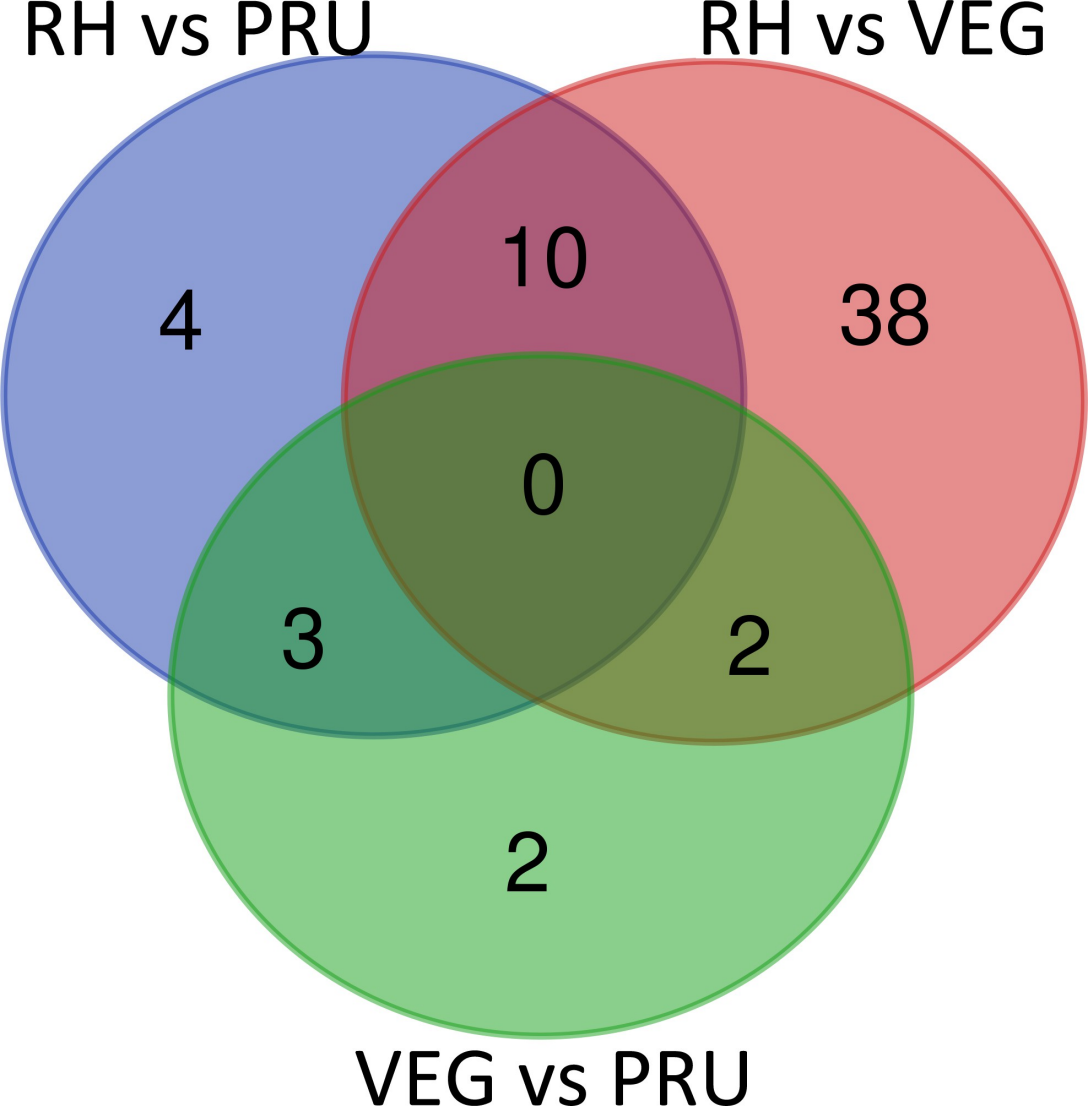
Venn diagram showing the unique and common differentially malonylated proteins in RH vs. PRU, RH vs. VEG and VEG vs. PRU.

Compared with the less-virulent PRU strain and the avirulent VEG strain, several malonylated proteins were down-regulated in the virulent RH strain. These included acetyltransferase, glyceraldehyde-3-phosphate dehydrogenase 2 (GAPDH2), arginyl-tRNA synthetase family protein, 3-ketoacyl-(acyl-carrier-protein) reductase, ATPase (DUF699) protein, cytochrome C, putative, ribosomal-ubiquitin protein, RNA recognition motif-containing protein, GNAT family protein, matrix antigen 1MAG1, and CDPK1 (Fig 10). Lysine acetyltransferase is involved in stage-specific gene expression and plays a role in *T*. *gondii* response to high pH (alkaline) [23]. Compared to the wild-type strains, RH strain lacking histone acetyltransferase is less sensitive to alkaline pH and exhibits low expression of stress-related genes [27].

The glycolytic enzyme GAPDH has two isozymes, with diverse activities and different subcellular localization in *T*. *gondii*. GAPDH2, located in the apicoplast of *T*. *gondii*, produces nicotinamide adenine dinucleotide phosphate (NADPH), which together with thioredoxin reductase and its substrate thioredoxin forms thioredoxin system [28], which plays a key role in the parasite intracellular survival [29]. *T*. *gondii* relies on thiol-reduction systems such as thioredoxin to counter oxidative stress and maintain parasite redox status [30]. Virulence factors, such as thioredoxin reductase protects *T*. *gondii* against oxidative-burst damage from host immune cells by catalysing the conversion of oxidized thioredoxin into its reduced redox state with the consumption of NADPH [31]. Maintaining this thioredoxin-reduction state enables the parasite to resist free radical injury in host immune cells. This result corroborates previous finding showing that control of intracellular *T*. *gondii* infection of naïve macrophages by type III, but not type I, depends on NADPH activity and elevated reactive oxygen species level, independent of interferon activation, indicating that the improved survivability and infectivity of the virulent *T*. *gondii* strains may be related to their ability to block reactive oxygen species production [32].

Aldehyde dehydrogenase, chaperonin, putative, glycosyl transferase, putative (predict) were the three most common down-regulated malonylated proteins in RH vs. PRU and VEG vs. PRU (Fig 10). Also, two common down-regulated malonylated proteins (glyceraldehyde-3-phosphate dehydrogenase 1 (GAPDH1) and phosphoglycerate kinase 1 (PGK1) were identified in RH vs. VEG and VEG vs. PRU (Fig 10). Both GAPDH1 and PGK1 are glycolytic enzymes located in cytosol and synthesize pyruvate from D-glyceraldehyde 3-phosphate. The pyruvate serves as a substrate for the pyrvuvate dehydrogenase to produce acetyl-CoA, required for the synthesis of fatty acids, which are critical for the parasite growth and proliferation [33]. The deletion of GAPDH1 causes sharp reduction in ATP levels in *T*. *gondii*, which was not compensated by GAPDH2 [34]. The enzymatic activities of GAPDH1 seems to be affected by other PTMs, with phosphorylation of the regulatory S-loop modulating glycolysis and palmitoylation regulating the association of GAPDH1 with the cortical membrane skeleton of *T*. *gondii* via Cys3 at the N-terminus [34]. To what extent malonylation of GAPDH1 contributes to the virulence and energy metabolism in *T*. *gondii* remains to be investigated.

Some of the differentially malonylated proteins between RH vs. PRU strains were mainly enriched in the regulation of actin cytoskeleton and actin binding. Likewise, in PRU vs. VEG strains the differentially malonylated proteins were enriched in actin binding. Furthermore, the upregulated proteins in VEG vs. PRU strains were enriched in actin cytoskeleton organization. Actin cytoskeleton plays a key role in the motility and invasion processes of *T*. *gondii* [35]. Toxofilin, an actin-binding protein secreted by *T*. *gondii*, facilitates the parasite invasion by dismantling the actin structure of the host cells; in the absence of toxifilin, an intact cellular actin cytoskeleton impedes *T*. *gondii* invasion [36].

In conclusion, differences in lysine malonylation between *T*. *gondii* strains representative of three main genotypes were determined using mass spectrometry and immuno-affinity purification. This analysis revealed many differentially regulated malonylated proteins in RH strain (type I), PRU strain (type II) and VEG strain (type III). Malonylated proteins were enriched in diverse enzymatic, biosynthetic, and metabolic processes. While our data indicate that lysine malonylation plays a role in the regulation of *T*. *gondii* virulence, the molecular details of the function and biological significance of the identified virulence related malonylated proteins remain to be unveiled. Nevertheless, the data obtained shed new light on the molecular mechanisms underpinning virulence differences between *T*. *gondii* strains with different genetic backgrounds and emphasize the impact of post-translational modification on the virulence of *T*. *gondii*.

## Supporting information

S1 Table The identified proteins in *Toxoplasma gondii* RH, PRU and VEG strains. (XLSX)

S2 Table The identified differentially malonylated proteins in *Toxoplasma gondii*. (XLSX)

S3 Table KEGG pathway enrichment of the differentially malonylated proteins in RH vs. VEG strains. (XLSX)

S4 Table GO analysis of the differentially malonylated proteins in PRU vs. VEG strains. (XLSX)
